# Learning can be detrimental for a parasitic wasp

**DOI:** 10.1371/journal.pone.0238336

**Published:** 2021-03-23

**Authors:** Valeria Bertoldi, Gabriele Rondoni, Ezio Peri, Eric Conti, Jacques Brodeur

**Affiliations:** 1 Dipartimento di Scienze Agrarie, Alimentari e Ambientali, Università degli Studi di Perugia, Perugia, Italy; 2 Dipartimento di Scienze Agrarie e Forestali, Università degli Studi di Palermo, Palermo, Italy; 3 Département de Sciences Biologiques, Institut de Recherche en Biologie Végétale, Université de Montréal, Montréal, Québec, Canada; Institut Sophia Agrobiotech, FRANCE

## Abstract

Animals have evolved the capacity to learn, and the conventional view is that learning allows individuals to improve foraging decisions. The parasitoid *Telenomus podisi* has been shown to parasitize eggs of the exotic stink bug *Halyomorpha halys* at the same rate as eggs of its coevolved host, *Podisus maculiventris*, but the parasitoid cannot complete its development in the exotic species. We hypothesized that *T*. *podisi* learns to exploit cues from this non-coevolved species, thereby increasing unsuccessful parasitism rates. We conducted bioassays to compare the responses of naïve *vs*. experienced parasitoids on chemical footprints left by one of the two host species. Both naïve and experienced females showed a higher response to footprints of *P*. *maculiventris* than of *H*. *halys*. Furthermore, parasitoids that gained an experience on *H*. *halys* significantly increased their residence time within the arena and the frequency of re-encounter with the area contaminated by chemical cues. Hence, our study describes detrimental learning where a parasitoid learns to associate chemical cues from an unsuitable host, potentially re-enforcing a reproductive cul-de-sac (evolutionary trap). Maladaptive learning in the *T*. *podisi*—*H*. *halys* association could have consequences for population dynamics of sympatric native and exotic host species.

## 1. Introduction

Animal decision-making, which is involved in processes such as resource and habitat selection, mate choice and progeny allocation, relies on innate behaviour (instinct), stochastic processes, physiological feedbacks (e.g., hormonal signalling) and learning (reviewed in [1]). Insect parasitoids have been used as model systems to explore both proximate and ultimate perspectives of optimal foraging. In order to cope with spatial and temporal variability in resources, parasitic wasps have evolved the capacity to associate host-related chemical cues to host availability and suitability [2–6]. They further consolidate and improve this capacity through learning processes [7–10], resulting in increased reproductive success [3, 11–13]. The probability of including a new stimulus in the behavioural repertoire of a parasitoid female depends on its reliability in host location [2], with oviposition having been shown to consolidate a change in foraging behaviour and host acceptance [3, 9, 14]. For example, scelionid parasitoids use host chemical cues on the egg surface or those deposited on plant surfaces by gravid females (footprints) to locate hosts in the habitat (reviewed by [15]). Following detection of host chemical cues, experienced scelionid females show stronger arrestment response (increased residence time, slower walking and increased turning tendency) than naïve females, a behaviour that is re-enforced by successful oviposition [16, 17].

Biological invasions generate novel interactions which can have negative consequences on populations of native species [18–21]. This occurs, for example, when an invasive exotic species becomes accepted as a host by native parasitoids but is unsuitable for offspring development. The introduced species then acts as an egg sink [22] for indigenous parasitoids, and negatively impact their reproductive success. We hypothesized that such an evolutionary trap [23, 24] could be exacerbated if foraging parasitoid females learn to exploit cues from a novel but unsuitable host. To our knowledge, there are no examples where associative learning actually results in costs to the foraging success of an animal.

To test this hypothesis we used *Telenomus podisi* Ashmead (Hymenoptera: Scelionidae), a common egg parasitoid of several North American stink bugs (Hemiptera: Pentatomidae) [25, 26]. As previously reported, *T*. *podisi* females accept and parasitize eggs of the brown marmorated stink bug, *Halyomorpha halys* Stål (Pentatomidae) [27], a recently established pest from eastern Asia [28], at a similar rate to that of its coevolved host, the predator *Podisus maculiventris* (Say) (Pentatomidae). But the parasitoid progeny rarely develop successfully in *H*. *halys* [27, 29–34], except for a nonconforming *T*. *podisi* population in California [35]. *Halyomorpha halys* is progressively spreading in invaded areas, thereby increasing the probability of native *T*. *podisi* encountering this novel but unsuitable host. We examined the learning capacity of *T*. *podisi* towards its coevolved host, *P*. *maculiventris*, and non-coevolved host, *H*. *halys*, and discussed consequences on interacting species.

## 2. Material and methods

### 2.1 Hosts and parasitoid

A colony of *H*. *halys* reared continuously on raw pumpkin seeds, carrots, green beans, grapes and potted soybean plants, was established from adults collected in Ontario (Canada) in 2012. The *P*. *maculiventris* colony was initially established using adults collected in Ontario in 2011 and 2012 and supplemented with bugs from Anatis Bioprotection (Canada). They were fed with live mealworm, *Tenebrio molitor* L. (Coleoptera: Tenebrionidae) larvae reared in the laboratory, fresh green beans and bean plants. Nymphs were kept in plastic cylinders and fed with mealworm and green beans. For both stink bug species, freshly laid eggs (< 24 h old) were used for the experiments.

The *T*. *podisi* colony was established with individuals collected in 2011 and 2012 in Ontario. Adult parasitoids were provided with a 1:1 (vol/vol) honey:water solution rubbed on a small piece of ParaFilm^®^. Each week, 1–2 days-old egg masses of *P*. *maculiventris* (stuck on filter paper using Pritt® stick glue) were exposed to *T*. *podisi* females for 24 h to maintain the colony. After emergence, male and female parasitoids were kept together in glass tubes for mating and provided with the honey-water solution. Naïve (i.e. without oviposition experience), 3–8 days-old females were randomly assigned to the different experimental treatments. Females *T*. *podisi* from our laboratory colony are synovigenic (ovigeny index < 0.05), typically emerging with very few mature oocytes (mean of approximately 2.0 [36]), and can survive for up to 100 days under laboratory conditions [37]. Unpublished data (M. Gaudreau, pers. com.) further show that 4–8 day-old females have an average of 14.2 mature eggs in their abdomen, similar to the maximum number of mature eggs observed per female throughout their reproductive life.

All insects were reared in a growth chamber (Conviron E15) at 24±1°C, 50±5 percent relative humidity, under a 16L:8D photoperiod.

### 2.2 Treatments

To determine if *T*. *podisi* females exhibit learning behaviour the following four treatments were tested: (i) naïve parasitoid females foraging on *H*. *halys* traces; (ii) females with experience on *H*. *halys* traces and eggs, then tested on *H*. *halys* traces; (iii) naïve females foraging on *P*. *maculiventris* traces; and (iv) females with experience on *P*. *maculiventris* traces and eggs, then tested on *P*. *maculiventris* traces. The experiments were conducted from 10:00 to 14:00 at 24±1°C, 50±5 percent relative humidity, under a 16L:8D photoperiod. Between 39 to 43 replicates were conducted for each treatment.

### 2.3 Obtaining experienced females

Experienced parasitoid females were obtained in the following manner. A female of either *H*. *halys* or *P*. *maculiventris* in their pre-ovipositional phase (with a physogastric abdomen) was introduced in an experimental arena consisting of a Petri dish (5 cm diam., 1 cm height) placed upside-down on a filter sheath. The Petri dish cover had tissue mesh (0.01 cm holes) to prevent saturation of the atmosphere with volatiles released by the stink bug female. Females walked for 30 minutes on the filter paper to allow contamination with chemical traces. Females that occasionally walked on the Petri dish were discarded. Filter papers soiled by bug’s faeces were discarded. Once the stink bug female was removed, a small egg mass (5–6 eggs) of the same species was placed in the middle of the contaminated area. A naïve (i.e. without foraging and oviposition experience) *T*. *podisi* female was then introduced in the arena and continuously observed. Once she had oviposited the female was removed, isolated in a 1.5 mL tube for 1 h before being tested as an experienced parasitoid. Females that did not oviposit within 1 h were discarded.

### 2.4 Parasitoid female response to chemical cues

As previously described [38], bioassays were conducted on a large filter paper arena (20 x 20 cm) where parasitoid females could move freely on the surface. A 5 cm diam area at the centre was exposed to a female of *H*. *halys* or *P*. *maculiventris* in pre-ovipositional phase, as described above. For the assays, a *T*. *podisi* female was released in the middle of the contaminated area (without host eggs) and her behaviour recorded with a HDD video camera (Sony HDR-XR 500) placed 40 cm above the arena. The assay stopped when the wasp left the arena or after 10 minutes [38]. Individuals that flew away within 10 s after the release were excluded from the analysis (n = 6). Each arena was used to test 5 females.

We recorded the time spent by the female in the arena (total residence time) and the number of times the parasitoids went back to the contaminated area once left (number of re-encounters), indicative of a tortuous path associated with the searching behaviour of a parasitoid female [15, 39, 40].

### 2.5 Statistical analyses

Generalized linear models (GLMs with Gaussian distribution for time data or Poisson distribution for count data) were fitted to test the effects of host herbivore species (*H*. *halys* or *P*. *maculiventris*), parasitoid previous experience (experienced or naïve) and their interaction on total parasitoid residence time and number of re-encounters with the contaminated area. Residence time data were subjected to Box-Cox transformation for normalization before the analyses. Significance of the model terms was evaluated by means of F test or Likelihood Ratio Test [41]. Significance of the different variable levels was assessed using the Tukey method for multiple comparisons procedure, adopting a significance level α = 0.05. The potential influence of chemical residuals from a parasitoid female on the behaviour of the following female was evaluated as a random effect in mixed models. This influence was negligible and therefore models with only fixed effect were included. Analyses were conducted under R statistical environment [42].

## 3. Results

**Fig 1:**
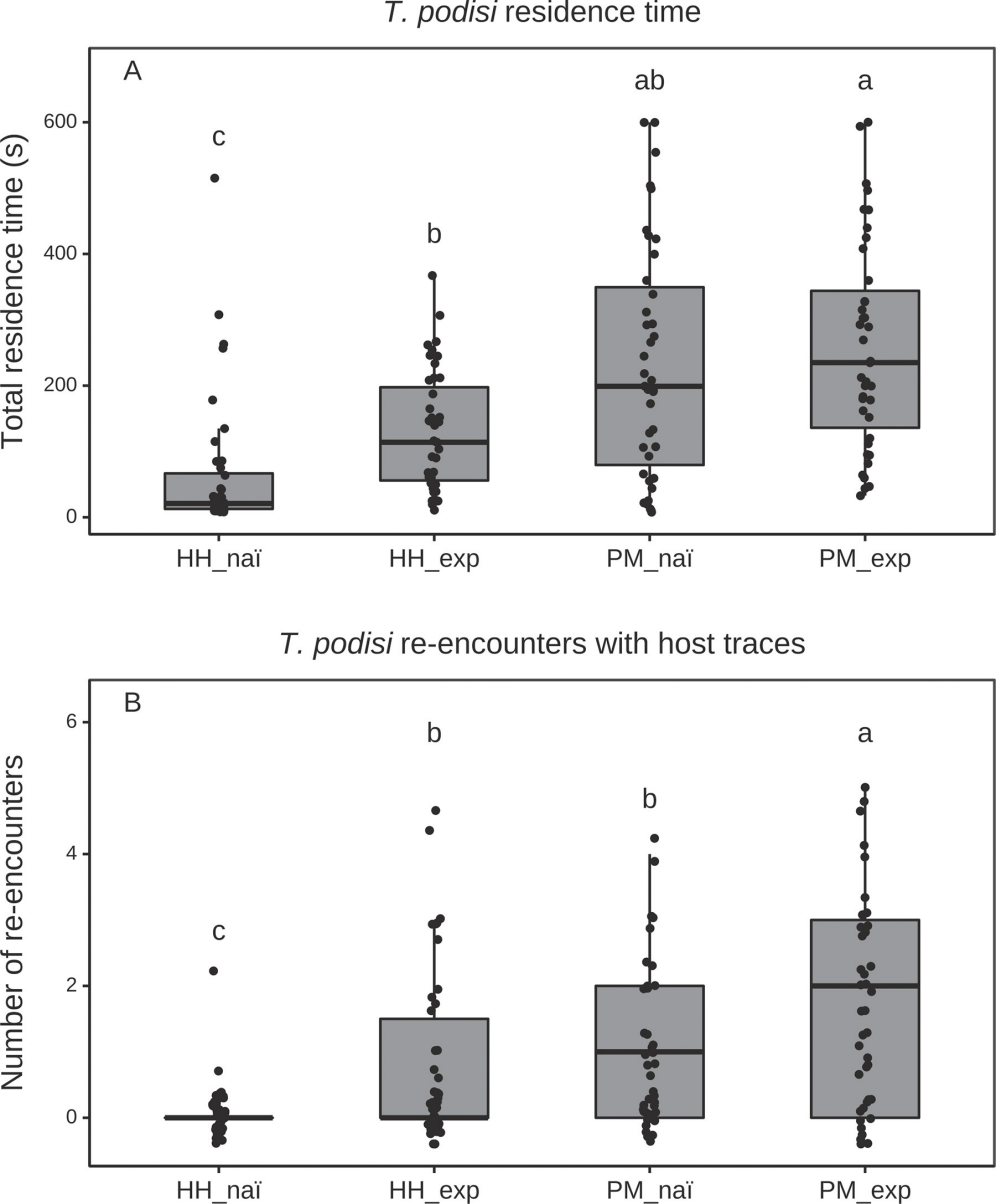
(A) Residence time and (B) number of re-encounters with the host-contaminated arena of naive and experienced wasps. The four treatments were: *T*. *podisi* naïve females tested on *H*. *halys* traces (HH_naï; N = 40); *T*. *podisi* females experienced on *H*. *halys* traces and eggs, then tested on *H*. *halys* traces (HH_exp; N = 43); naïve *T*. *podisi* females tested on *P*. *maculiventris* traces (PM_naï; N = 39); *T*. *podisi* females experienced on *P*. *maculiventris* traces and eggs, then tested on *P*. *maculiventris* traces (PM_exp; N = 39). Different letters above bars indicate significant differences between treatments (*p* < 0.05; GLM followed by Tukey method for multiple comparison procedure).

Total residence time of *T*. *podisi* varied depending on the insect species footprint (Gaussian GLM: F_(1, 159)_ = 53.78, P < 0.0001, the previous experience (F_(1, 158)_ = 16.83, P < 0.0001) and the interaction of these two factors (F_(1, 157)_ = 4.28, P = 0.04). For the *T*. *podisi—P*. *maculiventris* association, parasitoid females that had previously experienced the chemical traces left by their native host and had been rewarded with an oviposition did not have a greater residence time in the experimental arena than naïve females. In contrast, females with a rewarded experience on the exotic *H*. *halys* stayed significantly longer in the arena than naïve wasps when tested on chemical footprints of *H*. *halys* (Fig 1A). However, both naïve and experienced parasitoids displayed higher residence time on chemical traces of their coevolved host *P*. *maculiventris* than those of the exotic *H*. *halys* (Fig 1A).

The frequency of re-encounter with the area contaminated by chemical cues varied depending on the insect species footprint (Poisson GLM: X^2^ = 35.65, P < 0.0001), the previous experience (X^2^ = 26.39, P < 0.0001) and the interaction of these two factors (X^2^ = 12.3, P = 0.0005). Experienced *T*. *podisi* females re-entered the contaminated area more frequently than naïve females (Fig 1B). Naïve females tested on *H*. *halys* footprints rarely returned to the contaminated area. However, following a rewarded experience on *H*. *halys*, the number of re-encounters with this species was similar to the number of re-encounters of naïve females tested on *P*. *maculiventris* (Fig 1B). Both naïve and experienced *T*. *podisi* females returned more frequently to a patch contaminated by *P*. *maculiventris* than *H*. *halys* (Fig 1B).

## 4. Discussion

Our findings indicate that *T*. *podisi* females exhibit increased foraging behaviour following experience with chemical traces and oviposition in both its coevolved and novel host, probably due to associative learning [11, 43]. This capacity permits a female parasitoid to capture and retain information about host availability in the habitat and to adjust her foraging behaviour accordingly. However, the value of learning and its consequences on the reproductive success of *T*. *podisi* are opposite when exploiting a suitable (*P*. *maculiventris*) *vs*. unsuitable (*H*. *halys*) host. Exploiting *H*. *halys* eggs is maladaptive for *T*. *podisi* because it incurs significant costs to foraging females and leads to a cul-de-sac for their progeny [27]. *Telenomus podisi* females are not attracted to plants infested by *H*. *halys* under laboratory conditions [44], whereas *T*. *podisi* was the most common parasitoid sampled from *H*. *halys* eggs under field conditions in Canada [33]. *Halyomorpha halys* not only represents an egg sink for *T*. *podisi*, but also a ‘time sink’ (sensu [27]) since females increase time foraging in areas contaminated by the unsuitable host. The waste of time is further amplified when females protect the egg mass (patch guarding) from competitors and predators during several hours following oviposition [45].

Maladaptive learning has been reported in a number of social hymenopteran insects (bees and wasps) when copying the foraging decisions of conspecifics leads to the exploitation of already deprived food resources ([46] and references therein). It was also suggested [47] that new genetic combinations following hybridization in fishes and birds could negatively impact learning capacities, potentially leading to postzygotic reproductive isolation. Maladaptive learning has also been reported in parasitoid species used as biological control agents. When reared on alternative host species, artificial diets or artificial rearing units, parasitoids may partly loose their capacity to find and exploit the natural pest species [48, 49]. For example, it was shown [50] that prior experience of *Exeristes roborator* (Hymenoptera: Ichneumonidae) when reared in an artificial arena significantly altered the behavioural response of parasitoid females to their host, the European pine shoot moth, *Rhyacionia buoliana* (Lepidoptera: Tortricidae), when released in forests. The present study documents an original case of detrimental learning where a parasitoid learns to associate chemical cues from an unsuitable host, thereby re-enforcing a reproductive cul-de-sac (evolutionary trap).

Both naïve and experienced *T*. *podisi* females showed a higher response to chemical traces of *P*. *maculiventris* than of *H*. *halys*. This pattern was expected because derived stimuli from coevolved hosts are likely to evoke strong innate responses by naïve individuals; indeed the innate response can be stronger than the learned response in the location and acceptance of highly suitable hosts [51]. However, we cannot exclude an effect of the rearing host (*P*. *maculiventris*), as shown in other parasitoid species [52–54]. For instance, similar residence time between naïve *vs*. experienced wasps when exposed to *P*. *maculiventris* chemical traces could be partly explained by *T*. *podisi* females having already gained experience when developing in and emerging from *P*. *maculiventris* eggs.

Associative learning of cues from a novel but unsuitable host would also exacerbate the negative effects of the evolutionary trap on *T*. *podisi* populations. It may contribute to modify the community structure in areas invaded by *H*. *halys* through direct and indirect ecological effects [27, 55]. Recent exposure to *H*. *halys* may lead to an increase in *T*. *podisi*’s rate of parasitism on the invasive host (host switching), and a consequent decrease in parasitism of indigenous pentatomid species (apparent predation/parasitism; (+, -) type interaction). Additional research is required to determine the extent to which detrimental learning would affect the population dynamics of native stink bugs and parasitoids. We only tested females 1 h after their experience with host chemical cues and it would be important to determine how long they exhibit such learned behaviour under natural conditions, considering ecological factors such as the relative densities of native and exotic stink bugs and the persistence of the chemical cues in the footprints.

This original case of maladaptive learning arises from a situation where a native parasitoid encounters a new potential host species as a result of a biological invasion. On one hand, there is no operational ecological filter that stops host location and acceptance and, on the other hand, there is a strong physiological filter that prevents parasitoid development [27, 32]. Accordingly, a parasitoid could escape such an evolutionary trap by evolving (i) behavioural capacities to prevent acceptance of an unsuitable resource or (ii) physiological capacities to successfully reproduce in the novel host species [27, 56].

## Supporting information

S1 File R script. R scripts and supplementary figures.
(PDF)

S1 Data *T*. *podisi* data. (XLSX)
