# Supplementary material for: Learning can be detrimental for a parasitic wasp
Source: PLoS One. 2021 Mar 23;16(3):e0238336. doi: 10.1371/journal.pone.0238336 (PMC7987188; doi:10.1371/journal.pone.0238336)
Supplement: S1 File — R scripts and supplementary figures. (PDF) [file pone.0238336.s001.pdf]

R_script_T.podisi

This script, with processed data, was used to generate figures and statistics reported in Bertoldi et al., “Learning can be detrimental for a parasitic wasp”, submitted to Biology Letters

Load required packages; load and format the dataset

```
rm(list=ls())
library(knitr)
library(dplyr)
library(emmeans)
library(multcomp)
library(multcompView)
library(sciplot)
library(bestNormalize)
library(ggplot2)
dat <- read.csv("Tpodisi_data.csv", sep=";", header=T)
dat$comb <- factor(dat$comb, levels=c("HH_naï","HH_exp","PM_naï","PM_exp"))
```

Evaluate the effect of the host species and experience level on the total residence time

```
dat$y<-predict(bestNormalize((dat$tot+1),standardize=FALSE)$other_transforms$boxcox)
model <- glm(y ~ sp*tr, family=gaussian(link="identity"),data=dat)
modelr <- glm(y ~ 1, family=gaussian(link="identity"),data=dat)
ms<-anova(modelr,model, test="F")
ms$F[2] #F-value
```

```
## [1] 24.96143
```

```
ms$Pr[2] #Significance
```

```
## [1] 2.927245e-13
```

```
me <- emmeans(model, ~sp*tr)
kable(multcomp::cld(me, Letters=LETTERS, sort=TRUE),caption = "Total residence time of T. pod
isi in the different experimental treatments. Mean values followed by the same letter are not
significantly different for P = 0.05 according to GLMs with Gaussian distribution (on Box-Cox
tranformed response variable) followed by Tukey HSD multiple comparisons procedure.")
```

Total residence time of T. podisi in the different experimental treatments. Mean values followed by the same letter are not significantly different for P = 0.05 according to GLMs with Gaussian distribution (on Box-Cox tranformed response variable) followed by Tukey HSD multiple comparisons procedure.

	sp	tr	emmean	SE	df	asymp.LCL	asymp.UCL	.group
3	HH	naï	5.416239	0.4028551	Inf	4.626658	6.205821	A
1	HH	exp	7.870245	0.3885479	Inf	7.108705	8.631785	B

	sp	tr	emmean	SE	df	asymp.LCL	asymp.UCL	.group
4	PM	naï	9.238242	0.4079872	Inf	8.438602	10.037882	BC
2	PM	exp	10.029933	0.4079872	Inf	9.230293	10.829573	C

```
cat("  \n")
```

Fig. 1: box plot for total residence time

```
tiff(paste("Fig.1_Residence_time.tiff",sep=""), units = "in", width=8, height=0.6125*8, res=5
00)
bp <- ggplot(dat, aes(x = comb, y = tot, fill = "grey20"))
bp <- bp + geom_boxplot(notch = F, outlier.shape=NA, width=0.5)
bp <- bp + ylim(c(0,700))
my_colors <- c("#999999", "#999999","#999999","#999999")
bp <- bp + scale_fill_manual(values=my_colors)
bp <- bp + labs(title = italic("T. podisi")~ "residence time", x=" ", y = "Total residence ti
me (s)")
bp <- bp + theme_bw()
bp<-bp+ theme(panel.grid.major = element_blank(), panel.grid.minor = element_blank(),
panel.background = element_blank(), axis.line = element_line(colour = "black"))
bp <- bp + theme(legend.position="none")
bp <- bp + theme(axis.text.x = element_text(colour="black",size=13,angle=0,hjust=.5,vjust=.5,
face="plain"),
axis.text.y = element_text(colour = "black",size=11,angle=0,hjust=1,vjust=.4,face="plain"),
axis.title.x = element_text(colour="black",size=15,angle=0,hjust=.5,vjust=0,face="plain"),
axis.title.y = element_text(colour="black",size=15,angle=90,hjust=.5,vjust=2.5,face="plain"),
plot.title = element_text(size=18, face = "bold", hjust = 0.5))
comb.summarized <- dat %>% group_by(comb) %>% summarize(Max.tot=max(tot))
bp<-bp+geom_text(data=comb.summarized,aes(x=comb,y=50+Max.tot,label=c("c","b","ab","a")),vjus
t=0)
bp <- bp + geom_point(position = position_jitter(width = 0.03))
bp
dev.off()
```

```
## png
##   2
```

```
bp
```

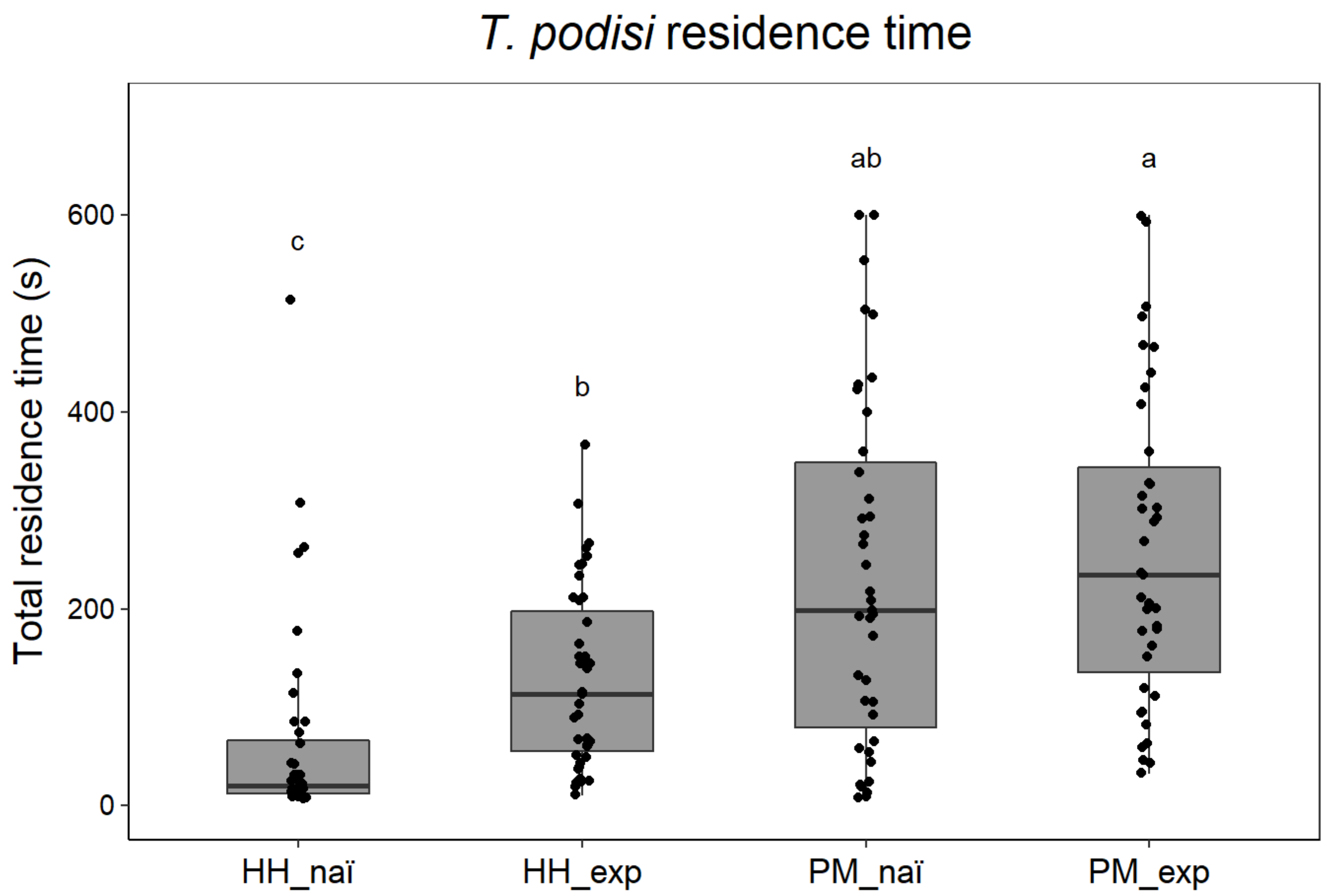

Evaluate the effect of the host species and experience level on the number of re-encounters

```
dat$y<-dat$re_enc
model <- glm(y ~ sp*tr, family=poisson,data=dat)
modelr <- glm(y ~ 1, family=poisson,data=dat)
ms<-anova(modelr,model, test="Chisq")
ms$Deviance[2] #Deviance difference
```

```
## [1] 74.34099
```

```
ms$Pr[2] #Significance
```

```
## [1] 5.015811e-16
```

```
me <- emmeans(model, ~sp*tr)
kable(multcomp::cld(me, Letters=LETTERS, sort=TRUE),caption = "Number of re-encounters of T.
 podisi with the contaminated area in the different experimental treatments. Mean values foll
owed by the same letter are not significantly different for P = 0.05 according to GLMs with P
oisson distribution followed by Tukey HSD multiple comparisons procedure.")
```

Number of re-encounters of T. podisi with the contaminated area in the different experimental treatments. Mean values followed by the same letter are not significantly different for P = 0.05 according to GLMs with Poisson distribution followed by Tukey HSD multiple comparisons procedure.

	sp	tr	emmean	SE	df	asymp.LCL	asymp.UCL	.group
3	HH	naï	-2.5902672	0.5773498	Inf	-3.7218519	-1.4586824	A
1	HH	exp	-0.1776812	0.1666666	Inf	-0.5043418	0.1489795	B
4	PM	naï	-0.0259755	0.1622214	Inf	-0.3439236	0.2919727	B
2	PM	exp	0.5411310	0.1221694	Inf	0.3016833	0.7805787	C

```
cat("  \n")
```

Fig. 2: box plot for number of re-encounters

```
tiff(paste("Fig.2_Re-encounters.tiff",sep=""), units = "in", width=8, height=0.6125*8, res=50
0)
bp <- ggplot(dat, aes(x = comb, y = re_enc, fill = "grey20"))
bp <- bp + geom_boxplot(notch = F, outlier.shape=NA, width=0.5)
bp <- bp + ylim(c(0,6.5))
my_colors <- c("#999999", "#999999", "#999999", "#999999")
bp <- bp + scale_fill_manual(values=my_colors)
bp <- bp + labs(title = italic("T. podisi")~ "re-encounters with host traces", x=" ", y = "Nu
mber of re-encounters")
bp <- bp + theme_bw()
bp<-bp+ theme(panel.grid.major = element_blank(), panel.grid.minor = element_blank(),
panel.background = element_blank(), axis.line = element_line(colour = "black"))
bp <- bp + theme(legend.position="none")
bp <- bp + theme(axis.text.x = element_text(colour="black",size=13,angle=0,hjust=.5,vjust=.5,
face="plain"),
axis.text.y = element_text(colour = "black",size=11,angle=0,hjust=1,vjust=0.4,face="plain"),
axis.title.x = element_text(colour="black",size=15,angle=0,hjust=.5,vjust=0,face="plain"),
axis.title.y = element_text(colour="black",size=15,angle=90,hjust=.5,vjust=2.5,face="plain"),
plot.title = element_text(size=18, face = "bold", hjust = 0.5))
comb.summarized <- dat %>% group_by(comb) %>% summarize(Max.re_enc=max(re_enc))
bp<-bp+geom_text(data=comb.summarized,aes(x=comb,y=0.7+Max.re_enc,label=c("c","b","b","a")),v
just=0)
bp <- bp + geom_point(position = position_jitter(width = 0.03))
bp
dev.off()
```

```
## png
##   2
```

```
bp
```

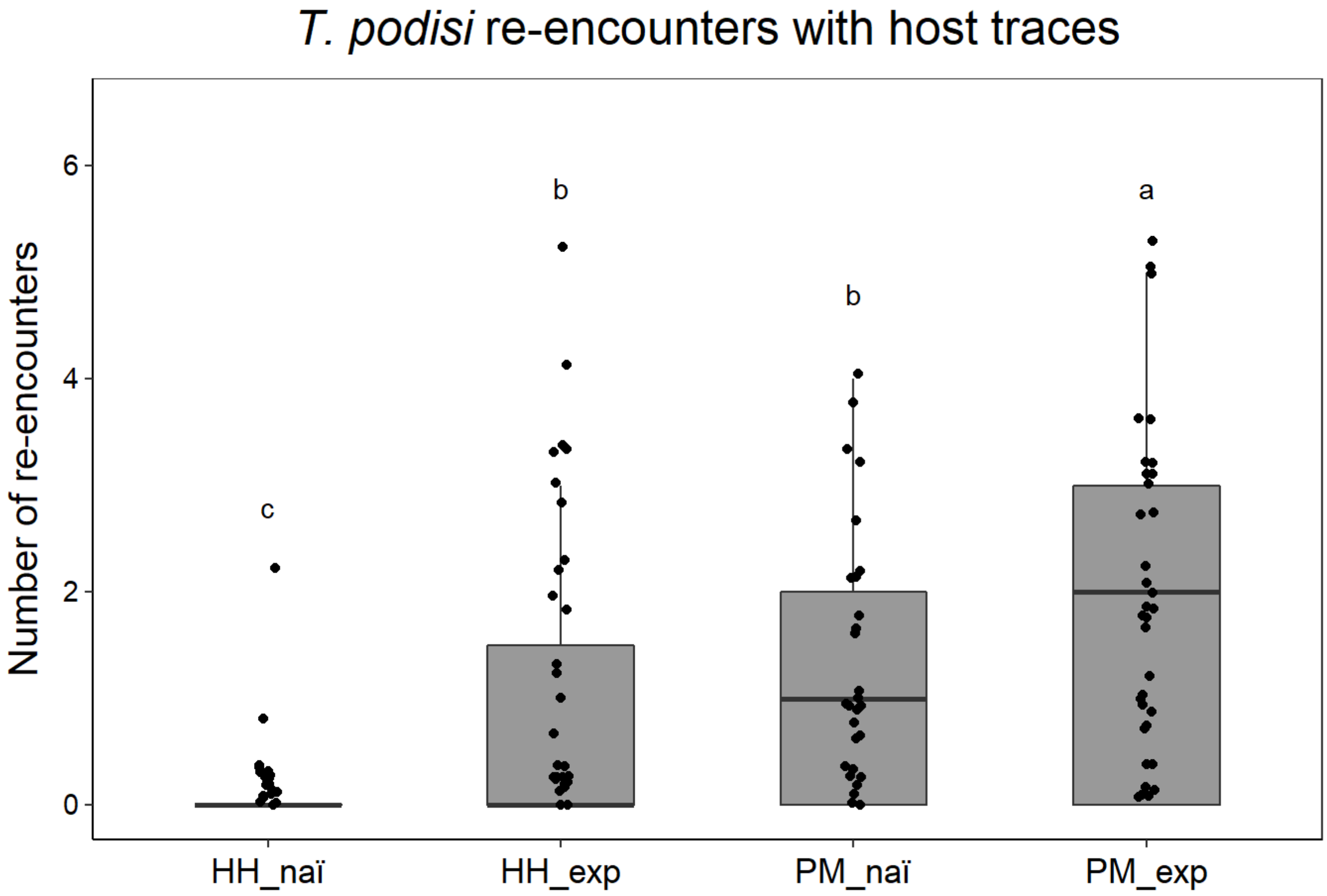

T. podisi re-encounters with host traces
Number of re-encounters
0
2
4
6
c
b
b
a
HH_naï
HH_exp
PM_naï
PM_exp
